# Case Report: IL-6 suppression: a potential strategy for mitigating severe immune-related adverse events in nivolumab and ipilimumab therapy for malignant pleural mesothelioma

**DOI:** 10.3389/fonc.2025.1671503

**Published:** 2025-10-31

**Authors:** Asako Yanagisawa, Takumi Fukaya, Kento Noda, Hiroshi Kojima, Hidefumi Shimizu

**Affiliations:** Department of Respirology, Japan Community Healthcare Organization (JCHO) Tokyo Shinjuku Medical Center, Tokyo, Japan

**Keywords:** malignant pleural mesothelioma, immune checkpoint inhibitor, nivolumab, ipilimumab, immune-related adverse Events (irAEs), IL-6, tocilizumab

## Abstract

**Background:**

Malignant pleural mesothelioma (MPM) is a rare and aggressive cancer with a poor prognosis, often presenting challenges in treatment. The CheckMate-743 trial demonstrated significant improvements in overall survival with the combination therapy of nivolumab and ipilimumab in advanced MPM. However, the management of immune-related adverse events (irAEs) remains a critical concern.

**Case presentation:**

We present a 74-year-old male with a history of polymyalgia rheumatica (PMR), diagnosed two years prior. His PMR was initially treated with corticosteroids, but tapering the dose was difficult. Therefore, tocilizumab was initiated one year before the current presentation, leading to a stable remission. One month before starting cancer therapy, tocilizumab was discontinued while the patient’s PMR remained well-controlled. He then developed multiple serious irAEs following the first cycle of nivolumab and ipilimumab for advanced MPM. These included cytokine release syndrome (CRS), immune-related aseptic meningitis, and liver dysfunction. All irAEs were successfully managed with corticosteroids, and the patient’s tumor progression remained under control.

**Conclusion:**

This case suggests that residual IL-6 suppression from prior tocilizumab therapy may attenuate the severity of subsequent irAEs, permitting effective management without compromising anti-tumor efficacy. IL-6 modulation could be a promising strategy to improve the therapeutic index of dual checkpoint inhibition in MPM.

## 1 Introduction

Malignant pleural mesothelioma (MPM) has a poor prognosis with a 5-year survival rate of less than 10% (1) before the introduction of immune checkpoint inhibitor (ICI) combination therapy. The results of CheckMate-743 trial demonstrated that nivolumab plus ipilimumab provided significant and clinically meaningful improvements in overall survival (OS) compared with standard-of-care chemotherapy (Median OS: 18.1 months [immunotherapy] vs. 14.1 months [chemotherapy]) (2). However, the management of immune-related Adverse Events (irAEs) remains a significant concern. Although it has been reported that irAEs occur more frequently in patients with autoimmune diseases and have a shorter time to onset (3, 4), it is unclear what adverse events may occur with immune checkpoint inhibitors in patients with autoimmune diseases in remission. Here, we report a case of MPM in a patient with pre-existing polymyalgia rheumatica (PMR) who developed multiple irAEs following nivolumab and ipilimumab therapy. We explore the potential role of residual IL-6 suppression from prior tocilizumab treatment in attenuating the severity of these irAEs, suggesting a novel perspective on managing ICI toxicity.

## 2 Case

A 74-year-old male was referred to our hospital with a one-week history of dyspnea on exertion, corresponding to grade 2 on the modified Medical Research Council scale.

He was diagnosed with polymyalgia rheumatica (PMR) at the age of 65. He was initially treated with prednisolone (30 mg/day) and showed a good response. However, his symptoms flared during the tapering of prednisolone. Consequently, tocilizumab was initiated, leading to prompt remission. At the age of 67, tocilizumab was discontinued, but it had to be restarted shortly thereafter due to a relapse of his symptoms. On presentation to our hospital, his PMR was well-controlled on a maintenance dose of prednisolone (2 mg/day) and ongoing tocilizumab therapy. He had a history of asbestos exposure between the ages of 22 and 27, and a 2-pack-year smoking history from age 20 to 50.

A chest X-ray (CXR) showed a right pleural effusion. Pleural fluid examination indicated increased mesothelial cells, but cytology was inconclusive. Considering the possibility of pleurisy caused by tocilizumab, which had been used for PMR, tocilizumab was discontinued.

The patient continued to have worsening pleural effusions. Subsequently a thoracoscopy examination under local anesthesia was performed.

The thoracic cavity was filled with a large amount of pleural fluid, and whitish nodular lesions on both the parietal and visceral pleura were observed.

Histopathological examination of biopsy specimens obtained from nodular lesions on the parietal pleura confirmed the diagnosis of malignant pleural mesothelioma. Furthermore, the hyaluronic acid level in the pleural effusion was found to be elevated.

**Figure 1:**
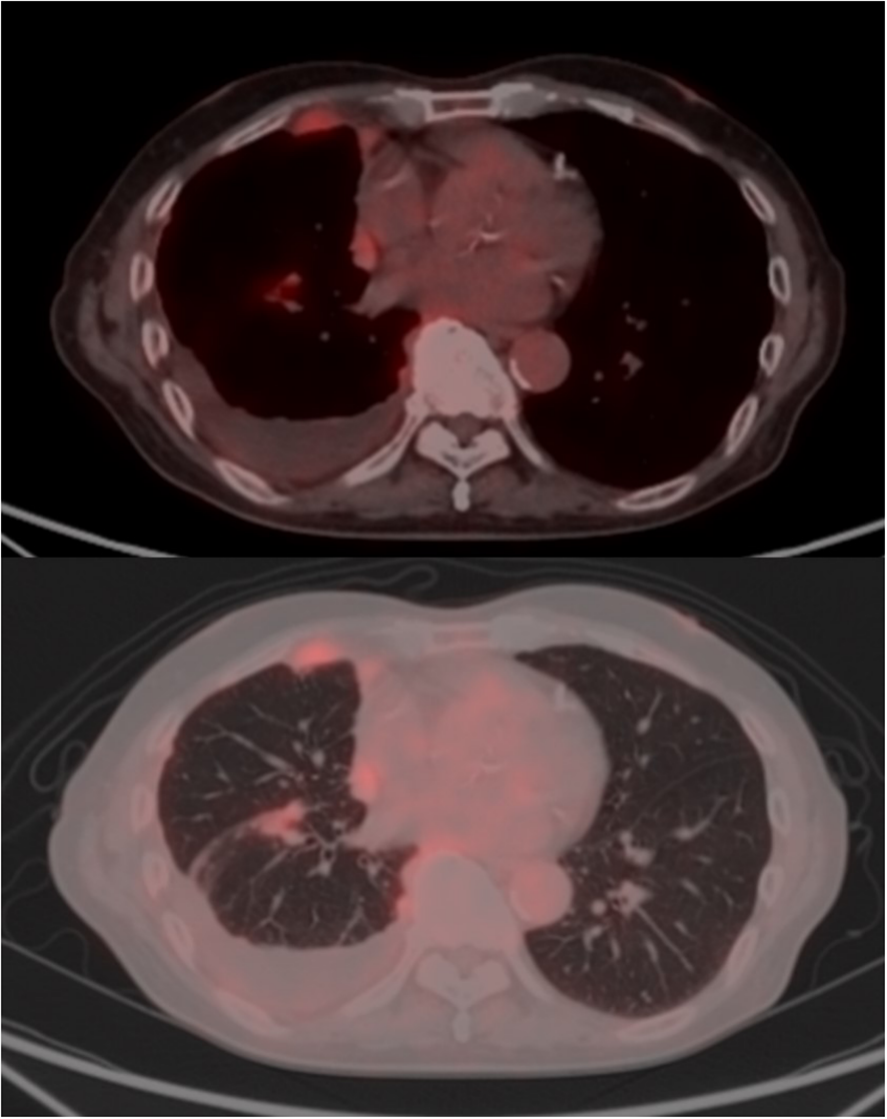
Radiological findings of malignant pleural mesothelioma. Positron emission tomography (PET) revealed asymmetric thickening of the right pleura, including the interlobar pleura, with heterogeneous fluorodeoxyglucose (FDG) uptake (peak SUVmax 4.0). Abnormal FDG accumulation was also observed in an enlarged lymph node-like structure located anterior to the right brachiocephalic vein and contiguous with the thickened pleura. Additionally.

Contrast-enhanced CT imaging for close examination revealed diffuse, slight pleural thickening with contrast enhancement. PET-CT revealed heterogeneous FDG uptake (SUV max 4.0) in the pleura, right paratracheal lymph node, and chest wall, including the interlobar pleura, which showed irregular thickening (**Figure 1**). Magnetic resonance imaging (MRI) of the brain indicated no metastatic lesion.

Based on these findings, the clinical staging was consistent with advanced unresectable MPM (cT4N1M0 stage III).

**Figure 2:**
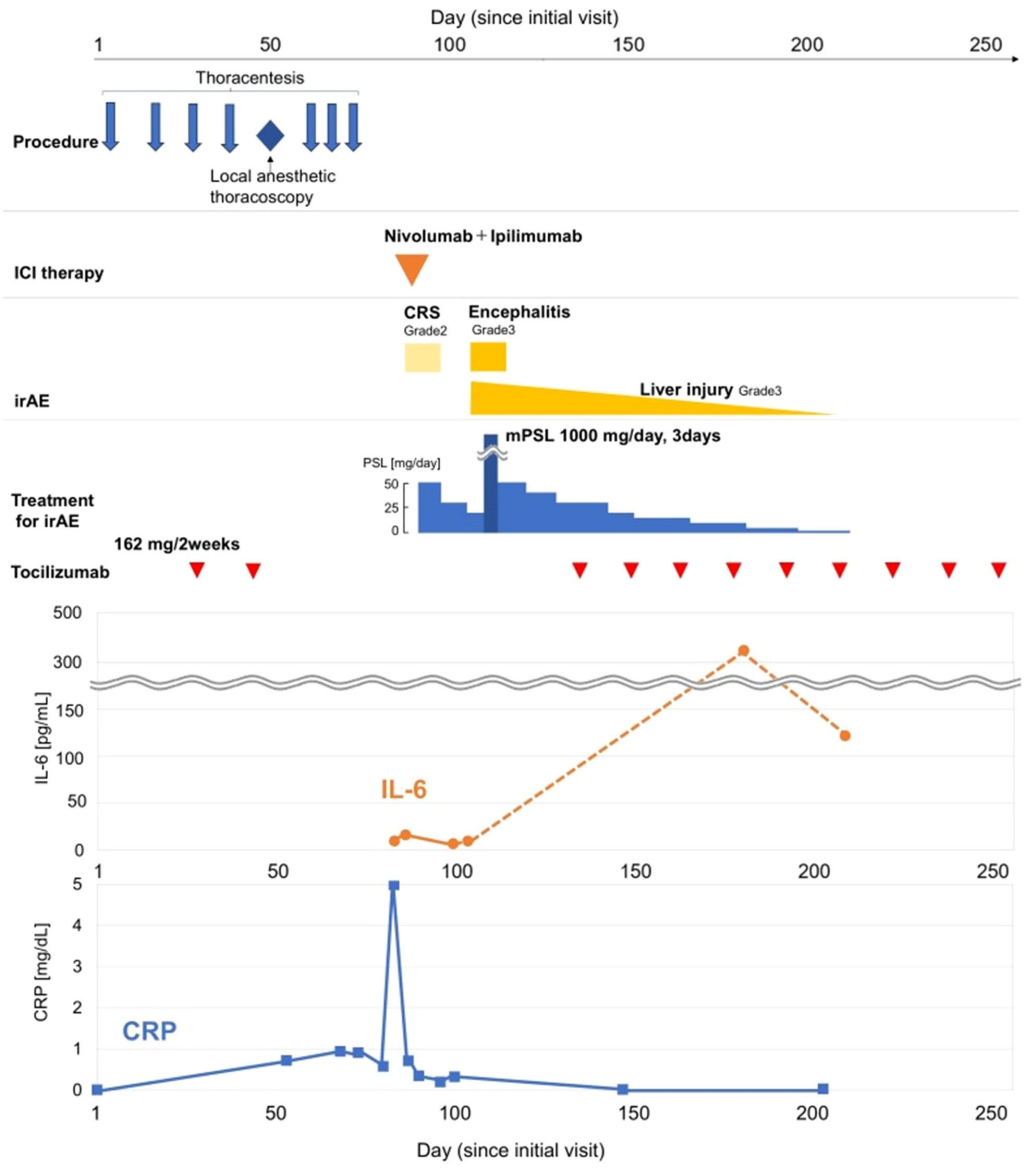
Timeline of the patient’s clinical course. The figure illustrates the clinical course of the patient, from diagnostic procedures to the management of immune-related adverse events (irAEs). The x-axis represents represents the number of days from the patient’s first visit (Day 0). The upper panels depict the timeline of procedures, the administration of nivolumab and ipilimumab on Day 82, and the subsequent onset, and treatment of irAEs, and the corresponding treatments. The lower panel shows the trends of C-reactive protein (CRP, blue line, right axis) and interleukin-6 (IL-6, orange line, left axis) levels.

Although the patient had previously experienced a relapse of PMR symptoms upon discontinuation of tocilizumab, no flare was observed this time. Therefore, we initiated first-line combination therapy with nivolumab and ipilimumab on day 82, one month after the last dose of tocilizumab (**Figure 2**).

The next day, he developed a fever and complained of fatigue, chills, and hypotension. Laboratory tests (**Table 1**) revealed an elevated C-reactive protein (CRP) level of 4.89 mg/dL, eosinophilia (13.0%, 670/μL), and hyponatremia (serum sodium 133 mmol/L).

**Table 1:** Key laboratory findings at baseline and during immune-related adverse events.

Category	Test	Reference	Unit	At baseline (day1)	At CRS onset (day 83)	At encephalitis onset (day 111)	After irAE resolution (day 204)
CBC	WBC	3.6-9.4	×10³/μL	8250	7140	7770	4600
	Hemoglobin	12.8 - 16.9	g/dL	11.9	13.2	13.7	12.1
	Hematocrit	39.7 - 50.8	%	36.7	39.4	40.7	35.9
	Platelet count	155 - 393	×10³/μL	34.5	31.7	18.1	21.5
Differential	Neutrophils	34.6 - 71.4	%	60.3	55.7	83.1	53.7
	Lymphocytes	19.6 - 52.7	%	23	19.2	9.5	28.5
	Monocytes	2.4 - 11.8	%	12.3	15.3	7.3	15.2
	Eosinophils	0 - 7.8	%	3.9	9.4	0	2.2
	Basophils	0 - 1.8	%	0.4	0.4	0.1	0.4
Biochemistry	Total Protein	6.7 - 8.3	g/dL	6.4	6.4	6.8	6.4
	Albumin	3.8-5 - 3	g/dL	3.3	3.2	3.7	4.2
	AST	10 - 40	U/L	23	34	356	97
	ALT	5 - 45	U/L	22	35	850	151
	LDH	100 - 225	U/L	143	204	270	170
	ALP	38 - 113	U/L	78	88	542	218
	Total Bilirubin	0.2 - 1.2	mg/dL	0.3	0.5	1.8	0.6
	BUN	7 - 22	mg/dL	18.1	13.5	30	24.5
	Creatinine	0.5 - 1.15	mg/dL	0.83	0.75	0.99	1.11
	Sodium (Na)	137 - 147	mEq/L	138	133	134	141
	Potassium (K)	3.5 - 5	mEq/L	4.9	4.4	4.2	4.0
	Chloride (Cl)	98 - 108	mEq/L	97	95	88	100
	Calcium	8.5 - 10.5	mg/dL	9.4	8.7	9.3	9.7
	CRP	0 - 0.299	mg/dL	0.58	4.89	0.323	0.014
	Glucose(fasting)		mg/dL	148	144	353	144
	IL-6	0 - 5.8	pg/mL	9.5	16.3	9.5	122

Data are shown for baseline (before immune checkpoint inhibitor [ICI] therapy), day 83 (at the onset of cytokine release syndrome), and day 111 (at the onset of encephalitis and liver injury). ALP, alkaline phosphatase; ALT, alanine aminotransferase; AST, aspartate aminotransferase; BUN, blood urea nitrogen; CBC, complete blood count; CRP, C-reactive protein; IL-6, interleukin-6; LDH, lactate dehydrogenase; WBC, white blood cell.

Adrenocorticotropic hormone (ACTH) and cortisol levels were within normal limits. The IL-6 level was mildly elevated relative to the pretreatment baseline (16.3 pg/ml at CRS onset vs. 9.5 pg/ml just before ICI initiation). After ruling out infection with negative blood cultures, these findings were consistent with Grade 2 cytokine release syndrome (CRS). Oral prednisolone at 50 mg/day was initiated on day 83.

He promptly improved clinically. Given the inadequate control of the patient’s diabetes, prednisolone was tapered relatively early at a rate of 10 mg every three days to reduce the risk of hyperglycemia, and the patient was subsequently discharged on day 93.

The patient was emergently readmitted ten days after discharge for a sudden onset of agitation, abnormal behavior, and psychomotor excitement. He was sedated with haloperidol and diazepam after physical restraint proved ineffective in controlling his severe agitation and restlessness.

On neurological examination, the patient’s Glasgow Coma Scale (GCS) score was E3V3M5. The GCS score, particularly the verbal component (V3), was likely influenced by sedation. His pupils were of normal size and symmetrically reactive to light. The oculocephalic reflex was intact (doll’s eye sign present), and his respiratory pattern was regular, indicating preserved brainstem function. Other focal neurological deficits, particularly those suggestive of classic limbic encephalitis, were not apparent given his limited ability to cooperate.

Laboratory tests showed increased white blood cell count (10820/μL), and significant liver damage, with increased AST (356 U/L), ALT (856 U/L), GTP (862 U/L), and ALP (542 U/L).

Brain MRI revealed T2/fluid attenuated inversion recovery (FLAIR) hyperintensities in the left temporal lobe and hippocampus.

A lumbar puncture was performed. Cerebrospinal fluid (CSF) analysis revealed an opening pressure of 13 cmH_2_O, a mononuclear-predominant pleocytosis with a cell count of 225/mm³, and an elevated protein level. The CSF glucose level was normal, and no bacteria were found in the fluid. The FilmArray CSF panel was negative, and serological tests for syphilis and HIV were also negative. Folate, vitamin B12, and thyroid hormone levels were within normal limits. Of the autoantibodies, only the anti-GAD antibody was routinely available for testing in our clinical setting; this was measured and later confirmed to be negative.

The electroencephalogram (EEG) was recorded on day 102 while the patient was sedated and unable to follow commands. The posterior dominant rhythm was absent, and the background was disorganized. Fast activity in the beta range was also absent. No sleep waves, such as spindles or vertex sharp waves, were observed. The background consisted of continuous, diffuse, low-amplitude slow activity in the delta range, which was symmetric and generalized. No epileptiform discharges, such as spikes or sharp waves, were identified either spontaneously or during photic stimulation. Overall, these findings were consistent with a severe diffuse encephalopathy.

The diagnosis of autoimmune encephalitis was established based on a constellation of findings, in accordance with the diagnostic criteria by Graus et al (5). The patient developed an acute neurological syndrome following ICI therapy, characterized by psychiatric symptoms and repetitive speech without clinical seizures. Key objective evidence included MRI findings in the temporal lobe, significant CSF pleocytosis, and EEG findings consistent with severe encephalopathy without epileptiform discharges. Importantly, infectious etiologies were rendered unlikely by negative CSF panels and serology.

Based on this diagnosis, the patient was started on steroid pulse therapy with intravenous methylprednisolone (1000 mg/day for 3 days).

The patient’s state of consciousness improved, and behavioral symptoms subsided promptly. Follow-up electroencephalography revealed no notable abnormalities.

Regarding liver dysfunction, autoantibodies associated with liver were negative, and abdominal CT showed only mild blunting of the hepatic margin without any apparent abnormalities in the biliary system. Autoimmune diseases were ruled out, and drug-induced liver injury due to nivolumab and ipilimumab was diagnosed. Liver enzymes rapidly decreased following the initiation of steroids.

Corticosteroids were subsequently tapered down by 10 mg per week up to 30 mg/day.

He was discharged on day 14 after readmission. CXR confirmed near resolution of pleural effusion.

Prednisone was tapered in 10 mg increments every 7 days while ensuring that there was no re-exacerbation of symptoms.

**Figure 3:**
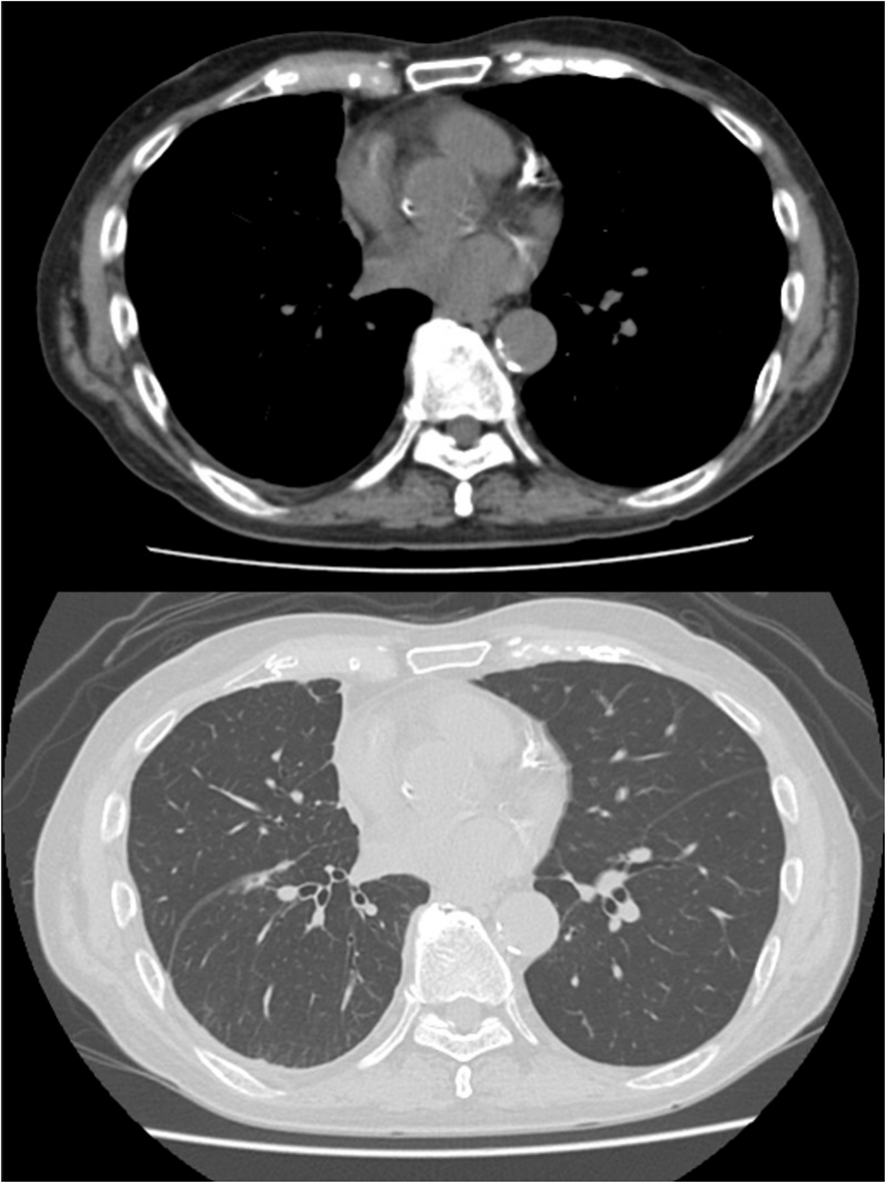
Radiographic response to a single cycle of immunotherapy. CT scans after one cycle of nivolumab and ipilimumab, demonstrating a significant reduction in the solid tumor lesions (white arrows) and complete resolution of the pleural effusion. CRP, C-reactive protein; CRS, cytokine release syndrome; ICI, immune checkpoint inhibitor; IL-6, interleukin-6; irAE, immune-related adverse event; mPSL, methylprednisolone; PSL, prednisolone.

Tocilizumab was reintroduced, after which no new immune-related adverse events were observed. The patient showed a remarkable response to just a single cycle of immunotherapy. His pleural effusion resolved completely, and the solid mesothelioma lesions also showed a durable reduction (**Figure 3**). As a result, he no longer required any pleural drainage that was necessary before treatment. The patient’s clinical condition remained stable, with no recurrence of pleural effusion or neurological symptoms until day 250. The overall clinical course of the patient, including therapies, adverse events, and key biomarker trends, is summarized in **Figure 2**.

## 3 Discussion

To our knowledge, this is the first report suggesting that residual IL-6 suppression from prior therapy may attenuate the severity of subsequent irAEs. While combination therapy with ICIs for MPM has demonstrated prolonged survival in MPM (6), the management of irAEs without compromising antitumor efficacy remains a major clinical challenge.

The rationale for integrating IL-6 inhibition with ICIs is twofold. First, IL-6 promotes tumor cell proliferation through STAT3 activation (7, 8). Second, clinical evidence indicates that tocilizumab can both alleviate irAEs and extend ICI treatment duration (9, 10). This strategy is particularly relevant in MPM, an inflammatory malignancy in which elevated IL-6 is associated with poor prognosis and systemic symptoms (11). Thus, targeting IL-6 in MPM may simultaneously mitigate irAEs and suppress tumor progression.

The present case was notable for the occurrence of multiple severe irAEs —including Grade 2 CRS, Grade 3 meningitis, and Grade 3 liver injury—all of which were rapidly controlled with corticosteroids. We hypothesize the residual effects of tocilizumab, administered one month prior for polymyalgia rheumatica (PMR), may have contributed to this favorable course. Tocilizumab’s long, half-life (12, 13)supports this interpretation, as does the observation that IL-6 levels were only mildly elevated during CRS (16.3 pg/mL vs. baseline 9.5 pg/mL) despite marked systemic inflammation (CRP 4.89 mg/dL). This attenuated IL-6 response suggests that persistent receptor blockade may have prevented a more refractory outcome.

Nevertheless, the complexity of cytokine networks warrants caution. IL-6 inhibition may trigger a compensatory upregulation of other cytokines, such as TNF-α, which can induce paradoxical inflammation. Clinically, new-onset psoriasis—a TNF-α–mediated disease—has been observed in patients treated with tocilizumab (14). This potential for TNF-α upregulation is a significant concern in oncology, as TNF-α is a potent activator of the NF-κB pathway, which is well-established to promote cancer cell proliferation (15). Fortunately, this pro-tumorigenic risk was not observed in our case. This successful management of a complex inflammatory state also supports the feasibility of ICI therapy in patients with controlled autoimmune disease. The patient’s PMR remained in remission, and all irAEs were manageable. Remarkably, a favorable clinical course has been maintained for six months following just a single administration of ICIs. This outcome is consistent with recent reports (16, 17) suggesting that ICIs can be administered safely in this setting.

In our case, tocilizumab was reintroduced after irAEs were controlled, leading to a rapid decline in CRP. The concurrent sharp increase in measured serum IL-6 is an expected pharmacological finding, reflecting receptor saturation by the drug rather than a loss of efficacy, thus confirming the effective inhibition of the IL-6 pathway (**Figure 2**).

In conclusion, this case highlights the potential role of IL-6 inhibition in controlling severe irAEs while preserving the antitumor activity of ICIs in MPM. With ongoing clinical trials on the combination of ICI and tocilizumab for malignant melanoma, urothelial carcinoma, and non-small cell lung cancer (NCT04940299), further studies are needed to clarify the optimal timing, dosing, and integration of IL-6 blockade in ICI-based regimens.

## Data Availability

The original contributions presented in the study are included in the article/supplementary material. Further inquiries can be directed to the corresponding author.
